# The role of family-related factors in the effects of the UP4FUN school-based family-focused intervention targeting screen time in 10- to 12-year-old children: the ENERGY project

**DOI:** 10.1186/1471-2458-14-857

**Published:** 2014-08-18

**Authors:** Wendy Van Lippevelde, Elling Bere, Maïté Verloigne, Maartje M van Stralen, Ilse De Bourdeaudhuij, Nanna Lien, Frøydis Nordgård Vik, Yannis Manios, Monika Grillenberger, Éva Kovács, Mai JM ChinAPaw, Johannes Brug, Lea Maes

**Affiliations:** Department of Public Health, Ghent University, De Pintelaan 185A, Watersportlaan 2, Ghent, 9000 Belgium; Department of Public Health, Sport and Nutrition, University of Agder, Postboks 422 N-4604, Kristiansand, Norway; Department of Movement and Sport Sciences, Ghent University, Watersportlaan 2, Ghent, 9000 Belgium; Department of Public and Occupational Health and EMGO Institute for Health and Care Research, VU University Medical Center, Van der Boechorstraat 7, Amsterdam, 1081 BT The Netherlands; Department of Nutrition, University of Oslo, Blindern, P.O. Box 1046, Oslo, 0316 Norway; Department of Nutrition and Dietetics, Harokopio University, 70 El. Venizelou, Kallithea, Athens 17671 Greece; Max Rubner-Institut, Federal Research Institute for Nutrition and Food, Haid-und-Neu-Str., Karlsruhe, 976131 Germany; Department of Paediatrics, University of Pécs, Vasvári Pál str. 4., Pécs, 7622 Hungary; Department of Epidemiology and Biostatistics and the EMGO Institute for Health & Care Research, VU University Medical Center, Van der Boechorstraat 7, Amsterdam, 1081 BT The Netherlands; Department of Public and Occupational Health and EMGO Institute for Health and Care Research, VU University Medical Center, Van der Boechorstraat 7, Amsterdam, 1081 BT The Netherlands

**Keywords:** Parents, Children, Screen time, Obesity prevention, Television, Computer

## Abstract

**Background:**

Screen-related behaviours are highly prevalent in schoolchildren. Considering the adverse health effects and the relation of obesity and screen time in childhood, efforts to affect screen use in children are warranted. Parents have been identified as an important influence on children’s screen time and therefore should be involved in prevention programmes. The aim was to examine the mediating role of family-related factors on the effects of the school-based family-focused UP4FUN intervention aimed at screen time in 10- to 12-year-old European children (n child–parent dyads = 1940).

**Methods:**

A randomised controlled trial was conducted to test the six-week UP4FUN intervention in 10- to 12-year-old children and one of their parents in five European countries in 2011 (n child–parent dyads = 1940). Self-reported data of children were used to assess their TV and computer/game console time per day, and parents reported their physical activity, screen time and family-related factors associated with screen behaviours (availability, permissiveness, monitoring, negotiation, rules, avoiding negative role modeling, and frequency of physically active family excursions). Mediation analyses were performed using multi-level regression analyses (child-school-country).

**Results:**

Almost all TV-specific and half of the computer-specific family-related factors were associated with children’s screen time. However, the measured family-related factors did not mediate intervention effects on children’s TV and computer/game console use, because the intervention was not successful in changing these family-related factors.

**Conclusion:**

Future screen-related interventions should aim to effectively target the home environment and parents’ practices related to children’s use of TV and computers to decrease children’s screen time.

**Trial registration:**

The study is registered in the International Standard Randomised Controlled Trial Number Register (registration number: ISRCTN34562078).

## Background

The prevalence of overweight and obesity in children has increased during the past decades and is associated with various physical (e.g., sleep apnoea, cardiovascular risk factors, type 2 diabetes) and psychosocial (e.g., social stigma linked to obesity) health problems in childhood [1]. Sedentary behaviours have been associated with obesity in childhood [2]. Sedentary behaviours can be defined as behaviours that require a minimal energy expenditure (1.0 – 1.5 metabolic equivalent units) and includes activities such as sitting and lying down [3]. A recent meta-analysis by Trembley and colleagues [2] showed that increased sedentary time was related to unfavourable body composition in 5- to 17-year-old boys and girls. Currently, evidence for a longitudinal positive relationship between sedentary time and body mass index (BMI) and more specific indicators of fat mass is insufficient [4]. Nevertheless, earlier studies have indicated that decreasing any type of sedentary time is associated with lower health risk in youth aged 5–17 years, independent from physical activity levels [2, 5]. Therefore, intervention programmes focusing on sedentary time are warranted.

The most prevalent sedentary behaviour in youth is electronic media use, especially TV viewing [6]. Recently developed guidelines on sedentary behaviour recommended that children should not spend more than two hours/day using TV/computer/electronic games [2]. However, mean levels of screen time in schoolchildren across Europe were found to exceed two hours per day [7, 8]. Between 25% and 40% of 11- to 15-year-old European and North-American children report watching TV more than three to four hours per day [7]. In addition, previous studies [8–10] have indicated that young adolescents played computer games for an average of 1–1.5 hours per day.

To date, several interventions that focus on reducing sedentary behaviours in general or specifically screen time have been developed, but their effectiveness on children’s screen time, sedentary time and BMI was small [11–13]. Salmon and colleagues [14] attributed this limited effect to setting-specific interventions (i.e., home-based, school-based or community-based), suggesting that intervening across multiple settings (e.g., school- and home-based) may be more effective in changing children’s sedentary behaviours. Furthermore, earlier research found that parental rules/restrictions of screen-based behaviours, the number of TVs in the home and parental role modelling of sedentary behaviours were the three most important correlates of screen time in 10- to 12-year-olds [15–19]. Studies examining correlates of screen time in children and adolescents have focused predominantly on TV viewing [14]. These factors indicate the significance of targeting the family in developing interventions that prevent sedentary behaviour.

The UP4FUN intervention is a school-based and family-focused pilot intervention aimed at reducing and breaking up sitting time at home and school in 10- to 12-year-old children in five European countries (Belgium, Germany, Greece, Hungary, Norway). This pilot intervention and its preliminary evaluation were part of the “EuropeaN Energy balance Research to prevent excessive weight Gain among Youth” (ENERGY)-project, a European Commission-funded project that aimed to develop and evaluate a theory-informed and evidence-based multi-component obesity prevention intervention for 10- to 12-year-old schoolchildren across Europe [20]. Unpublished findings by Vik and colleagues on the short-term effectiveness of the UP4FUN intervention suggest that the intervention was not effective in reducing total TV time and computer/game console use. Nevertheless, earlier research highlights the importance of conducting theory-driven mediation analysis of intervention data to understand the underlying mechanisms of change in sedentary behaviours, even if there is no intervention effect [14, 21–23]. Mediation analyses can provide useful insights in the underlying working mechanisms of interventions even without significant intervention effect. For example, an intervention can effectively influence the presumed mediator if the mediator is not related to the outcome, which indicates that the presumed mediator is not a good predictor of the outcome. Intervention developers can subsequently decide to remove the intervention component targeting this presumed mediator. Alternatively, the presumed mediator may be associated with the outcome, but the intervention did not affect the mediator. In this case, other intervention strategies targeting this mediator are required [22]. Additionally, mediation of non-significant intervention effects is possible when inconsistent mediation occurs, namely inconsistent mediators can suppress the intervention effects [21, 22].

Van Stralen and colleagues [23] conducted a literature review on mediators of overweight prevention interventions in children and adolescents and found only three school-based studies [24–26] that explored the mediators of interventions focusing on sedentary behaviours. Potential family mediators included were screen time rules and social norm but no significant mediators were found. Nevertheless, the authors suggested to further explore mediation effects of environmental variables (including parental factors) on sedentary behaviours, as these behaviours may not be well-considered, conscious behaviours but instead be influenced by individual biological factors, habit strength and parental factors [16, 23].

The purpose of the present study was to (i) examine the effects of the UP4FUN intervention on family-related factors, (ii) investigate associations between changes in these family-related factors associated with TV and computer/game console time and changes in children’s screen time (i.e., TV and computer/game console time), and (iii) determine the role of family-related factors in the UP4FUN intervention effects on TV and computer/console time in 10- to 12-year-old children.

## Methods

### Design and participants

This pilot intervention was tested in the autumn of 2011 using a pre-test post-test design including an intervention and a control condition in five European countries. The intervention condition included a six-week school-based family-focused intervention, and the control condition involved the usual school curriculum. A convenience sample of schools was recruited in each country and schools were paired according to size. Subsequently, schools were randomly allocated to the control or intervention group. In total 62 schools participated in the study with 31 intervention schools and 31 control schools. Study participants were 10- to 12-year old children and one of their parents. All participating countries obtained ethical approval from the relevant ethical committees and ministries. The study was approved by the Medical Ethics Committee of the University Hospital Ghent in Belgium; by the State Medical Chamber of Baden-Württemberg in Germany; by the Bioethics Committee of Harokopio University in Greece; by the Scientific and Ethics Committee of Health Sciences Council in Hungary; and by the National Committees for Research Ethics in Norway. The participating parents in all countries except Belgium provided their written consent through their child’s consent form, thereby also agreeing to the participation of one of the child’s parents. In Belgium, the participating parents consented by returning the parent questionnaire as passive informed consent were allowed by the ethics committee. The study is registered in the International Standard Randomised Controlled Trial Number Register (registration number: ISRCTN34562078; http://www.controlled-trials.com/isrctn/). More extensive information about the design and participants is reported elsewhere [27].

### Intervention

A school-based family-focused pilot intervention focusing on reducing children’s sedentary behaviours in school and at home was developed and implemented over a six-week period.

### Theoretical framework and behaviour change techniques

The development of the UP4FUN intervention was based on the five steps of the Model of Planned Promotion of Population Health [28] and the Intervention Mapping protocol [29]. Changing personal determinants of sedentary time (i.e., awareness, attitudes, preferences, self-efficacy) was considered important to promote self-regulation, because 10- to 12-year olds are likely to spend a considerable amount of non-supervised time at home. The intervention was also framed in a social ecological perspective [30] because of the clear influence of the physical and social home environment [19]. The taxonomy of behaviour change techniques [31] was applied to characterise the association between the determinants and intervention components (Table 1). The taxonomy of behaviour change techniques and intervention components was based on information gathered from systematic reviews, secondary data-analyses, focus group research and findings from stakeholder interviews about intervention ideas.

**Table 1:** The UP4FUN intervention by social ecological level, determinant, behaviour change techniques* and program components

Social ecological model level	Determinant	Behaviour change techniques	Examples of program components
**Individual**	Knowledge	Provide information about behaviour – health link (IMB)**	Week 1 (sedentary/screen time), Week 5 (breaking up sitting time), Family Fun Event (week 6), Website
	Attitude	Prompt intention formation (SCogT)**	Week 1: Pupils start wearing the UP4FUN bracelet
	Awareness of own behaviour	Prompt self-monitoring of behaviour (CT)**	Week 2:
			• Pupils draw timeline with activities for a normal week day
			• Pupils register steps for 3 every day activities at home
			• Pupils register sitting time by activity (TV, PC, games and reading) for one weekday and one weekend day
		Provide feedback on performance (CT)**	Week 3:
		Prompt specific goal setting (CT)**	• Pupils sum up sitting time and write personal goal (NEWS 3) and are provided with information on recommendations about duration of screen time
		Provide contingent rewards (OC)**	• Pupils try out the goal for a week, evaluate with stickers (Smileys and Frownys) and write down 3 difficulties and solutions
	Automaticity	Teach to use prompts or cues (OC)	Week 5: Activity breaks in class, make poster(s) of things to do during breaks
		Prompt practice (OC)	NEWS 5: Ideas for how to remember to do breaks at home during PC/TV time
			Week 5: Teacher reminds pupils to get out quickly for recess.
	Preference/ liking		Week 2: Pupils make a list of 3 indoor and 3 outdoor fun activities to do at home
			Week 5:
			• Class brainstorms ideas for recess activities and makes a poster of them
			• Pupils register steps for walking to school
			• Pupils try out activity breaks in school
	Self-efficacy	Prompt barrier identification (SCogT)	Week 3:
			• Pupils try out the goal for a week and write down 3 difficulties and solutions
			• Class discusses difficulties and solutions
			Week 4: Optional: Family has screen free day + write 3 positive and negative experiences
	Role modelling	Prompt identification as role models (SCogT)	WEEK 5 : Pupils encouraged to model activity breaks during TV-time for family
**Interpersonal (family)**	Knowledge	Provide information about behaviour – health link (IMB)	NEWS 1 (sedentary/screen time), NEWS 5 (breaking up sitting time), Family Fun Event (Week 6), Website
	Awareness of child behaviour	Prompt monitoring of child behaviour (CT)	NEWS 2: Pupil share drawing of when they sit/what they do during a regular week day. Parents are encouraged to take notice when their child is sitting,
			NEWS 3: Pupils share results of sitting registration time and personal goal with parents
	Social support	Plan social support and social change (social support theories)	NEWS 2: Pupils share list of 3 indoor and 3 outdoor fun activities to do at home with family
		Agree on behavioural contract (OC)	NEWS 3: Pupils share results of sitting registration time and personal goal with parents, and are encouraged to ask for help from family members. Suggestion for parents on the different types of support they can offer and how to communicate.
		Plan social support and social change (social support theories)	
			NEWS 5: Suggestions: Plan for active transport
			Week 6/NEWS 6:
			• Family participates in Family Fun Event (results from the project, sharing of positive and
			• negative experiences, practicing Activity breaks, take on the Family challenge = to continue to work on reducing sitting time)
			• Teacher hands out bracelets to families that take the challenge
	Role modelling	Prompt identification as role models (SCogT)	NEWS 4: Suggestions: Positive parental modelling and doing fun alternatives together or change the physical availability of screens
	Parenting rules and restrictions	Provide opportunities for social comparison (SCompT)**	NEWS 4: Pupils share with parents the number of pupils in class with rules about screen time and some examples of the rules, and discuss family screen rules Suggestions: Choose one rule and try it out for a week.
	Physical availability of screens	Prompt barrier identification (SCogT)**	NEWS 4: Pupils and then parents guess number of screens at home by category (TV, PC, games) before pupils count them. Suggestions: Change the physical availability of screens.
**Organizational (teacher)**	Knowledge	Provide information about behaviour – health link (IMB)	Teacher training, teacher manual, Website
	Role modelling	Model or demonstrate the behaviour, Teach to use prompts or cues (OC)**	Week 5: Teacher modelling of activity breaks once in every sitting lesson, suggested to use alarms as reminders

*for definitions of behaviour change techniques see Abraham and Michie [31].

**IMB = information-motivation-behavioural skills model CT = control theory OC = operant conditioning SCogT = social-cognitive theory SCompT = Social comparison theories.

At the individual level, the following behaviour change techniques were applied to target the determinants knowledge, attitude, awareness, automaticity, preference, self-efficacy, and role modelling of the pupils: information about the behaviour-health link, self-monitoring with normative feedback on behaviour and goal setting with self-rewards, use of prompts/prompt practice and identification as role models. The main targets at the interpersonal level were the parents, but the children could define who in their home should help them. They were also encouraged to ask for and offer help to their peers. Planning for social support and social change was therefore the most used change technique at the interpersonal level, but the following additional techniques were used to target parents’ knowledge, awareness of child behaviour, role modelling, rules and restrictions and physical availability of screens at home: information about the behaviour-health link, agreement on a behavioural contract, monitoring of child behaviour, opportunities for social comparison and identification as role models. Finally, at the organisational level, the teacher was provided information about the behaviour-health link and trained to model breaking-up sitting time and to use prompts and cues to remember to do so. In Table 1, the practical application of these behaviour change techniques in the UP4FUN intervention can be found. Additionally, pretesting was conducted in all five countries to test the core intervention components and identify any substantial cultural differences. More extensive information about the underlying theoretical frameworks and the pretesting phase are described in Lien and colleagues [27].

### Description of the UP4FUN intervention program

The name UP4FUN was chosen to remind the pupils to get UP and engage in FUN alternatives to sitting. A design company developed the material so that it should appeal to children of both genders across Europe through a general youth culture image, and inspire to activity and fun without promoting organised sports. The intervention included 6 phases (6 weeks) with 1–2 lessons per phase/week (Table 2). These lessons introduced the topic of the phase, followed by tasks in school, messages to the family and tasks at home in the 6 newsletters to parents/family. Each newsletter contained 2–3 pages and was designed to transfer the children’s personalised messages from school to home (e.g., about the results of their sitting time registration and their personal goal for change) and to work on the topic of the phase through tasks at home. During the intervention, motivating factors (economic incentives) were used to support the fun part of the intervention (i.e., step counters and stickers) and the social commitment to the message (i.e., bracelet with UP4FUN embossed). The program ended with a Family Fun Event in phase 6. The aim of this event was to summarise the project, share experiences and raise continued support for the main message. Alternatively, this concluding event could be done in class. Newsletter 6 aimed to convey the main results from this event (Table 2 details the six phases). More information about the intervention can be found elsewhere [27].

**Table 2:** **The UP4FUN intervention - the phases, the NEWS and the tasks**

Phases and titles		NEWS titles	Tasks
1	Introduction to UP4FUN	NEWS 1: *Welcome!*	- Families talk about the project at home
			- Families note down time for Family Fun Event
			- Pupils start wearing the UP4FUN bracelet
2	Awareness of sitting time and light physical activity alternatives.	NEWS 2: *Awareness of time spent sitting.*	- Pupils draw timeline with activities for a normal week day and share it with family
			- Pupils make a list of 3 indoor and 3 outdoor fun activities to do at home and share the list with family
			- Pupils register steps for 3 every day activities at home
			- Pupils register sitting time by activity (TV, PC, games and reading) for one weekday and one weekend day
3	A challenge – reducing sitting time	NEWS 3: *Helping and supporting your child to aim for less sitting time.*	- Pupils sum up sitting time
			- Pupils write personal goal
			- Pupils share results of sitting time and personal goal with family
			- Pupils try out the goal for a week, evaluate with stickers (Smileys and Frownys) and write down 3 difficulties and solutions
			- Class discusses difficulties and solutions
4	Home environment and sitting time	NEWS 4: *Do screens control your family life?*	- Pupils note down number of pupils in class with rules about screen time and some examples of the rules, share this with parents and discuss family screen rules
			- Pupils and then parents guess number of screens at home by category (TV, PC, games) before pupils count them
			- Suggestions: Reduce parental modelling and family screen time or change the physical availability of screens
			- Optional: Family has screen free day + write 3 positive and negative experiences
5	Breaking up prolonged sitting time and practicing active transport	NEWS 5: *Short activity breaks are better than no breaks at all.*	- Class brainstorms ideas for recess activities and makes a poster of them
			- Teacher leads one Activity break per sitting lesson throughout the week. Pupils practice at home.
			- Pupils practice active transport to school and register the number of steps
			- Pupils remind parents about Family Fun Event
6	Summarizing the class results and spreading the Challenge.	NEWS 6: *Thank you for taking part in the UP4FUN project.*	- Class prepares the Family Fun Event
			- Family participates in Family Fun Event (results from the project, sharing of positive and negative experiences, practicing Activity breaks, take on the Family challenge of continuing to work on reducing sitting time)
			- Teacher hands out bracelets to families (parents and siblings) that take the challenge either at the Family Fun Event or after a response to NEWS 6

### Measurements

Measurements were conducted according to standardised protocols [27], and data were collected before (September/October 2011) and after the intervention (November/December 2011). The children completed questionnaires during school time and received the parent questionnaire in a closed envelope to take home for completion by one of their parents.

### Child screen time (TV and computer time)

Time spent on TV viewing and computer/console use was assessed separately by two questions asking the children how many hours they usually spend watching TV and using computer/console on a weekend and weekday, respectively. The outcome variable “TV viewing” included watching all TV programs and films (also DVD) on a TV or on a computer”. Computer/console” use was defined as playing games on a computer, games console or mobile phone and using the Internet for leisure activities such as chatting, e-mailing, surfing, and Facebook.

Average TV and computer/console time per day was calculated by multiplication of the number of days per week and screen time per day in hours divided by 7.

### Parental measures (demographics, family-related factors)

In the parent questionnaire, demographics, self-reported levels of screen time and other family-related factors associated with screen time were assessed.

Age, weight, height, sex, and educational level of both parents were asked in the parent questionnaire. Educational level was categorised as ‘none of the parents had education for 14 years or more’ and ‘one or both parents had education for 14 years or more’ , which approximately distinguishes between families with at least one parent or caregiver with medium-level vocational training or higher education and families in which both parents were lower educated.

Questions about screen time on the parental questionnaire were similar to those in the child questionnaire; screen use was assessed by frequency questions referring to a usual day.

The following family factors (i.e., the hypothesized mediators) were assessed in the parental questionnaire: availability of screens, strictness/permissiveness, monitoring, rules, avoiding being a negative role model, physical activity and sport behaviour of parent, and active family trips. Table 3 shows the exact formulations of the family-related questionnaire items and their psychometric characteristics. The items were based on and informed by the Pro Children and ENDORSE parent questionnaires [32, 33]. The items had a five-point answering format. Exploratory factor analyses showed that some items could be collapsed into the following subscales: ‘strictness related to screen time’ , and ‘active family trips’ (Table 3). The subscales and other singular family-related items were used as mediator variables in the model.

**Table 3:** **The most relevant questionnaire items and their psychometric characteristics**

Factor	Question item	Response alternatives	Cronbach’s alpha
Availability TV’s	How many TV’s do you have at home?	0 = none – 5 = more than 4	
Availability computers	How many computers do you have at home (including iPads and laptops)?	0 = none – 5 = more than 4	
Availability consoles	How many game consoles do you have at home (e.g. Playstation, Xbox, Nintento, Wii, GameCube, DS)?	0 = none – 5 = more than 4	
Strictness (2 items)	1. When my child asks me if he/she can watch TV /use the computer/console during leisure, I allow it	-2 = always – +2 = never	0.58
	2. My child can watch TV/use computer/game console during leisure whenever he/she wishes		
Paying attention/Monitoring	I pay attention to how much time my child spends watching TV/using a computer/game console during leisure	-2 = never – +2 = always	
Negotiating	I discuss with my child how long he/she can watch TV/use computer/game console during leisure	-2 = never – +2 = always	
Rules	1. I have rules about how many hours my child can watch TV/use a computer/game console during leisure	-2 = fully disagree – +2 = fully agree	
Avoiding negative role modeling	When I want to watch TV/use the computer/game console during leisure, I will hold back when my child is around	-2 = never – +2 = always	
Parental PA	During an average week, on how many days have you been physically active for at least 30 minutes in total	0 = none – 7 = every day	
Parental sporting	How often do you do sports? (e.g. fitness club, indoor or outdoor)	0 = never – 4 = more than once a week	
Active family trips (3 items)	1. How often do you and your child go on a bike-or-hike trip in the neighbourhood together?	0 = never – 4 = more than once a week	0.74
	2. How often do you and your child go on a nature excursion together (e.g. woods, mountains, lake, river, sea)?		
	3. How often do you and your child go on other nature related trips together (e.g. to the park)		

### Validity and reliability of the measurements

Used items were based on valid and reliable items from the earlier ENERGY-cross-sectional study, the validity and reliability of these items were tested in a study among 10- to 12-year-old children (n = 730 in the test-retest reliability study; n = 96 in the construct validity study) and one of their parents (n = 316 in the test-retest reliability study; n = 109 in the construct validity study) in six European countries. The item of TV time in the child questionnaire had a good test-retest reliability (ICC = 0.68) and construct validity (0.70), and the computer item had a moderate test-retest (ICC = 0.54) but a weak construct validity (ICC = 0.28). More information can be found in Singh et al. [34]. Concerning the family factors related to screen time, all items showed good to excellent test-retest reliability as indicated by ICCs > 0.66. Construct validity was moderate to excellent for all items except one, as indicated by ICCs > .51. More information can be found in Singh et al. [35]. Additionally, a test-retest reliability study was conducted on the UP4FUN child and parent questionnaires. A convenience sample of six schools was selected in autumn 2011: one school in Belgium, four schools in Hungary and one school in Norway, including 143 pupils and 105 parents. The test-retest reliability showed good values for the items included in this paper (ICCs ranged from 0.51 to 0.80) [36].

### Statistical analyses

Preliminary descriptive analyses of sample characteristics were conducted using SPSS (version 21). A complete cases design was used for conducting the analyses for both behaviours. Only children who had valid measurements for TV and computer time at both pre- and post-test, respectively, and whose parents completed the questionnaire at pre- and post-test were included.

Residualised change scores of screen behaviours (TV and computer/game console time) were computed by regressing screen time scores at post-test onto screen time scores at pre-test. The resulting residualised scores can be interpreted as the change in screen time between pre- and post-test, adjusted for pre-test scores. Similarly, residual change scores of the family-related items were calculated. The residuals were checked for normality and were considered acceptable.

Multilevel linear regression analyses (2-level: children within schools) were conducted using MLwiN version 2.22 to provide answers to the three research questions. To assess mediating effects, the product-of-coefficient test of MacKinnon was used [21]. The first step in the mediation analyses was to investigate the intervention effect on the outcome variable (c-path). However, the main effect of the intervention on both screen behaviours (TV and PC/console use) was already described by Vik et al. (unpublished data). The second step was to estimate the effect of the intervention on changes in the potential mediator (Action theory test: a coefficient). The third step was to estimate 1) the independent association of changes in the potential mediator and changes in the outcome controlling for the effect of the intervention (Conceptual theory test: b coefficient); and 2) the effect of the intervention on changes in the outcome variable controlling for changes in the potential mediator (c’-path). To represent the mediated effect, the product of the two coefficients (a coefficient*b coefficient), was calculated [21]. The statistical significance of the mediated effect was estimated by dividing the product-of-coefficient (a*b) by its standard error. For the calculation of the standard error the Sobel test was used (SE_ab_ = √(a^2^*SE_b_^2^ + b^2^*SE_a_^2^). Significance was set at the p < 0.05 level. All analyses were adjusted for the children’s age, children’s gender, parents’ years of education and country, as these constructs were significantly associated with the outcome variables. Additionally, the need for country-specific mediation analyses was checked by examining the moderating role of country on the intervention effects. In models with the different family-related variables and TV and computer/game console time used separately as the outcomes, a test for interaction between country of residence and the intervention was conducted. However, no country-specific analyses were conducted as country was not a significant moderator of the intervention effect on screen time.

## Results

### Sample characteristics

In total, 3325 children and 2945 parents completed the pre-test questionnaire in the five countries, from which 1949 and 1940 children and parents, respectively, had valid data for both the pre and post-test measurements of TV and computer/game console time and family factors. Characteristics of the pre-test sample are shown in Table 4.

**Table 4:** **Characteristics of the European children in the intervention and control group at pretest (September 2011)**

	Baseline sample	
	Intervention group	Control group
	n = 1569	n = 1578
**Demographics**	M ± SD	M ± SD
Age child	11.3 ± 0.8	11.2 ± 0.8
Age parent	40.6 ± 5.1	40.4 ± 5.2
Sex (%)		
Female	49.7	52.7
Sex parent (%)		
Female	85.9	88
Family educational level		
at least one parent ≥14 years of education	62.8	63.7
**Behaviours**		
Children’s TV viewing (hours/day)	1.6 ± 1.0	1.6 ± 1.0
Children’s computer/game console (hours/day)	1.2 ± 1.0	1.2 ± 1.0
Parents’ TV viewing (hours/day)	2.1 ± 0.9	1.6 ± 1.0
Parents’ computer/game console (hours/day)	0.6 ± 0.7	0.6 ± 0.7

### Mediation analyses

#### Intervention effect (c-path)

As reported by Vik et al. (unpublished), no significant intervention effects were found for total TV and computer/game console time (in the current dataset: c_pre-post TV time_ = -0.020(0.026), p = 0.37; and c_pre-post Computer time_ = -0.029(0.036), p = 0.38). No country-specific analyses were conducted as country was not a significant moderator of the intervention effect on screen time.

### Action theory test (a coefficient)

The intervention did not lead to significant changes in any of the family-related factors related to either TV or computer/game console time (Table 5). No country-specific analyses were conducted, as country was not a significant moderator of the intervention effect on the family-related variables.

**Table 5:** **Action and conceptual theory test, and mediation effects of the family-related factors on the UP4FUN intervention effect (conducted in 2011 among European children)**

Family-related factors	a	b	ab
*Outcome: TV time*			
Parental TV time	-0.017(0.039)	0.197(0.020)**	-0.003(0.008)
TVs at home	-0.036(0.057)	0.066(0.015)*	-0.002(0.004)
Permissiveness TV viewing (2 items)	0.025(0.03)	-0.200(0.023)**	-0.005(0.006)
Paying attention/monitoring TV viewing	-0.017(0.019)	-0.254(0.028)**	0.004(0.005)
Negotiating TV viewing	0.048(0.026)	-0.095(0.021)*	-0.005(0.003)
Rules TV viewing	0.014(0.029)	-0.117(0.021)*	-0.002(0.003)
Avoiding negative TV role modeling	-0.004(0.026)	-0.081(0.025)	0.000(0.002)
Parental PA week	-0.019(0.081)	0.008(0.010)	0.000(0.001)
Parental sports	0.044(0.047)	-0.066(0.016)*	-0.003(0.003)
Active family trips (3 items)	0.023(0.028)	-0.086(0.025)	-0.002(0.002)
*Outcome: computer/game console time*			
Parental computer/console behaviour	-0.004(0.018)	0.280(0.034)**	-0.001(0.005)
Number of computers	-0.059(0.056)	0.030(0.015)	-0.002(0.002)
Number of consoles	-0.041(0.051)	0.057(0.014)*	-0.002(0.003)
Permissiveness computer/console use (2 items)	0.028(0.024)	-0.188(0.027)**	-0.005(0.005)
Paying attention/monitoring computer/console use	0.009(0.017)	-0.190(0.035)*	-0.002(0.003)
Negotiating computer/console use	0.062(0.027)	-0.032(0.023)	-0.002(0.002)
Rules computer/console use	0.03(0.024)	-0.042(0.025)	-0.001(0.001)
Avoiding negative computer/console role modeling	0(0.027)	-0.047(0.024)	0(0.001)
Parental PA week	-0.017(0.08)	0.026(0.011)	0.000(0.002)
Parental sports	0.038(0.044)	-0.051(0.016)	-0.002(0.002)
Active family trips (3 items)	0.02(0.026)	-0.003(0.027)	0.000(0.001)

*p < 0.05, **p < 0.01.

c- coefficient: estimate of the intervention effect on residualized change score of outcome behaviour.

c’-coefficient: estimate of the intervention effect on change score of outcome behaviour, adjusted for changes in family environmental factor.

a-coefficient: effect of intervention on residualized change score of family-environmental factor.

b-coefficient: estimate of the independent effect of the change in mediator on residualized change score for outcome behaviour, adjusted for total effect from intervention on outcome behaviour.

ab product-of-coefficient estimate; mediated effect.

Three-level regression models were conducted: children nested within schools within countries, all regression models were adjusted for children’s age, children’s gender, and parents’ years education.

Countries included in the study: Belgium, Germany, Greece, Hungary and Norway.

### Conceptual theory test (b coefficient)

Changes in almost all family-related factors related to TV time, except for avoiding negative role modelling, parental physical activity (PA) and family trips, were significantly associated with changes in children’s TV time. Positive associations with TV time were found for parental TV time and number of TVs at home. In contrast, parental monitoring, negotiating, rules and strictness, and sport behaviour were inversely related to TV time. Four of the 11 family-related factors were significantly related to children’s computer/game console time. Parental strictness and monitoring were negatively associated with computer/game console time. Parental modelling, and availability of consoles at home were positively related to children’s screen time (Table 5).

### Mediated effects

None of the examined family-related factors showed a mediating effect on changes in screen time (Table 5). This result was because the intervention had no significant effect on these family factors.

## Discussion

The six-week UP4FUN pilot intervention is one of the first interventions aimed at reducing and breaking up sitting time in children both at school and home through a school-based and family-focused prevention programme. The present study explored if changes in the family factors mediated the UP4FUN intervention effects on TV and computer/console time in 10- to 12-year-olds in Europe.

Notwithstanding the systematic and careful development based on theory, explorative research using stakeholders, and earlier experimental evidence, the UP4FUN intervention did not result in significant changes in the screen time of the children (unpublished data). Additionally, the present study has observed that no intervention effects were found on the family-related factors (i.e., the hypothesised mediators). This failure of intervention effects on the family factors may have several causes. First, this study was a pilot test of only six weeks with the main aim to test the feasibility of the intervention. Second, the family intervention may not have been adequately implemented in the intervention schools. Based on the process evaluation results, the family component did not reach a substantial number of parents [37]. Third, the insignificance of the intervention might be due to the family intervention components and/or strategies not being appealing/motivating, effective or extensive enough to change family-related factors despite the strategies being based on focus group research with parents of 10- to 12-year-olds conducted as part of the ENERGY-project. This qualitative study assessed parents’ perspectives about parental participation in school-based obesity prevention [38]. However, these focus groups also indicated that involving and motivating parents is difficult, so even if an intervention is able to reach the parents, they are not always eager to participate actively. The preliminary process evaluation supported this hypothesis, as not all parents read the newsletters despite receiving them, and few parents participated in the family fun event [37]. More experimental research is needed to find effective strategies to target the home environment [39]. A fourth explanation for the non-significance of the action theory could be that measures of the family-related factors were not sensitive enough to measure change [21, 40].

Despite the lack of an intervention effect on screen time and the family-related factors, significant associations of changes in family factors and changes in children’s screen time were found. Changes in four family-related factors (parental modeling, monitoring, strictness, availability of TVs and game consoles) were significantly associated with changes in both children’s TV and computer/game console time, which indicated their importance as potential determinants of children’s screen time. These results support earlier findings that indicate associations between greater screen time in children and greater parental screen-related behaviours, more sedentary opportunities at home (e.g., number of TVs, computers, games), and less rules/restrictions related to screen time [15–18]. Therefore, our study adds longitudinal evidence to these associations and consequently affirms that changes in the family factors and home environment are indeed important for reducing screen time in school-aged children. Future interventions focusing on reducing screen time should therefore include effective strategies targeting these factors. Whereas school-based interventions appear to be the default choice [41, 42], we may need to reconsider and explore better ways to include parents and target the home environment. The recent community-based interventions aiming to improve energy balance-related behaviours and reduce obesity risk in children may be a way forward [43].

To our knowledge, this is one of the first sedentary behaviour intervention studies that intervenes both at school and home, as well as one of the first examining family factors as mediators of a school-based, family-focused sedentary behaviour intervention in children. Moreover, in contrast to earlier studies focusing on associations between family-related factors and screen behaviours (e.g., mainly TV viewing), a large range of family-related factors were investigated for both TV viewing and computer/game console use separately. Therefore, this study also adds to the current literature information concerning relations between the home environment and children’s screen time. Another strength of this study was the large sample of children and parents from multiple European countries that participated in the pre- and post-test assessing sedentary behaviours and their determinants.

A few limitations of the study need to be mentioned as well. First, screen time and family-related factors were based on self-report and therefore might be answered to in a socially desirable way. Moreover, to limit the burden on the participants, single items to measure the family-related factors were used which could increase measurement error. Nevertheless, the included measures showed good test-retest reliability. Second, because convenience samples of schools in the participating countries were recruited, study results of the pilot intervention study cannot be generalised. Another limitation is the unequal distribution in the participation of mothers and fathers in the UP4FUN study. Only a small amount of participating parents were fathers. Earlier studies underline the limited available paternal data [44, 45]. Additionally, given the low child–parent agreement in reporting health behaviours and their determinants, the inclusion of both parents of a family - if feasible - may increase the measurement quality [44].

## Conclusion

The UP4FUN pilot intervention was not effective in reducing screen time in children or in changing family-related factors. Nevertheless, significant associations were found between changes in almost all TV-specific family-related factors and half of the computer/console-specific family-related factors and changes in children’s TV and computer/console use. These findings imply that future screen time interventions should aim strongly at parental practices and the home environment.

### Trial registration

The study is registered in the International Standard Randomised Controlled Trial Number Register (registration number: ISRCTN34562078; http://www.controlled-trials.com/isrctn/).
